# An Update on Umbilical Cord Abnormalities and Associated Thrombosis: A Systematic Review

**DOI:** 10.3390/clinpract16050092

**Published:** 2026-05-14

**Authors:** Marco La Verde, Eleonora Braca, Antonio Cerillo, Maria Fatigati, Pasquale De Franciscis, Davide Pisani, Mario Fordellone, Mariavictoria De Simone, Carlo Pietro Campobasso, Alessandro Feola

**Affiliations:** 1 Department of Woman, Child and General and Specialized Surgery, Obstetrics and Gynecology Unit, University of Campania “Luigi Vanvitelli”, 80138 Naples, Italy; eleonorabraca9@gmail.com (E.B.); antoniocerillo@gmail.com (A.C.); mara95f@hotmail.it (M.F.); pasquale.defranciscis@unicampania.it (P.D.F.); davidepisani94@gmail.com (D.P.); 2 Medical Statistics Unit, University of Campania “Luigi Vanvitelli”, 80138 Naples, Italy; mario.fordellone@unicampania.it; 3 Department of Experimental Medicine, University of Campania “Luigi Vanvitelli”, 80138 Naples, Italy; mariavictoriadsu@gmail.com (M.D.S.); carlopietro.campobasso@unicampania.it (C.P.C.); alessandro.feola@unicampania.it (A.F.)

**Keywords:** thrombosis, umbilical artery thrombosis, umbilical vein thrombosis, umbilical cord abnormalities

## Abstract

**Background and objective**: Umbilical cord abnormalities (UCAs) such as hypercoiling, velamentous or marginal cord insertion, or reduced Wharton’s jelly are associated with umbilical thrombosis. UCAs increase the risk of vascular obstruction and impaired fetal blood flow, resulting in hypoxia or stillbirth. We examined the association between the UCAs and the umbilical cord thrombosis. **Methods**: According to PRISMA, five electronic databases (PubMed, Scopus, Embase, Cochrane Library, and Clinicaltrial.gov) were screened. Only studies that analyzed umbilical cord insertion abnormalities and abnormalities of the umbilical cord associated with thrombosis were included in this systematic review. Studies without thrombosis were excluded (PROSPERO ID: CRD420251087525). **Results**: Only 12 articles out of 1105 screened records satisfied the inclusion criteria, comprising 3 retrospective cohort studies, 3 case series and 6 case reports. The publication years ranged from 1983 to 2025. A total of 126 cases of umbilical vascular thromboembolism (UVTE) were identified, among which 84 cases of UCAs represented by 16 cases of stricture, 14 cases of hypercoiling, 16 cases of too-short cords (≤40 cm), 11 cases of too-long cords (≥70 cm), 5 cases of velamentous or furcate cord insertions, 12 cases of nuchal cord insertions, 13 cases of funistisis, 11 cases of true knots, and 3 cases of Wharton jelly abnormalities. **Conclusions**: UCAs, including true knots, abnormal coiling, and furcate or velamentous cord insertion, were highly associated with UVTE. Future studies should involve developing standardized criteria for the diagnosis and reporting of UCAs.

## 1. Introduction

The umbilical cord connects the placenta to the fetus, and mediates oxygen and nutrient transfer essential for fetal growth [1]. Various structural and functional abnormalities of the umbilical cord are well-documented in adverse pregnancy outcomes [1]. Structural anomalies (e.g., knots, entanglements, abnormal coiling, or thrombosis) have been associated with impaired fetal blood flow [2].

Several umbilical cord abnormalities (UCAs) have been associated with stillbirth. Proposed mechanisms range from mechanical compression to vascular complications such as umbilical artery thrombosis (UAT) and venous thrombosis (UVTE) [3]. These complications can be due to maternal factors, such as gestational diabetes, thrombophilia, autoimmune disorders, or intrinsic cord abnormalities [4,5].

Despite these mechanisms, UCAs are often underdiagnosed prenatally [3,6]. This implies delayed intervention and adverse fetal outcomes [7]. Knowledge of these factors is important for improving diagnosis and management protocols, reducing stillbirth rates, and trying to close the gaps in current best practices pertaining to perinatology [8].

Structural UCAs could possibly relate to the umbilical thrombosis, particularly with structural anomalies (e.g., hypercoiling, velamentous or marginal cord insertion, or diminished Wharton’s jelly) [9]. These abnormalities increase the vascular obstruction, with severe consequences that include intrauterine growth restriction, hypoxia, and stillbirth [10,11]. According to Virchow’s hypothesis, the triad of risk factors for thrombosis is mainly represented by blood stasis, endothelial dysfunction, and hypercoagulability [12]. Although the pathogenesis of thrombosis is well known, UAT or UVTE represent a catastrophic intrauterine event, whose prenatal diagnosis remains a challenge [13]. Current diagnostic protocols are mainly based on routine ultrasonography, which has limitations in the detection of subtle thrombotic events or cord pathology, usually identified in the postnatal period [6]. Data on UCAs and their association with uterine activity remain limited [14,15].

Improved understanding of thrombosis associated with UCAs [9] and better prenatal detection strategies are needed [16]. We assessed the relationship between UCAs and umbilical thrombosis (UAT or UVTE) with emphasis on etiology, clinical presentation, and diagnostic challenges.

## 2. Materials and Methods

### 2.1. Eligibility Criteria

We included studies evaluating UCAs in association with umbilical vascular thrombosis. UCAs were defined as macroscopic or microscopic abnormalities of cord morphology, length, coiling, insertion, compression, inflammatory involvement, or Wharton’s jelly, including hypercoiling/hypocoiling, true knots, strictures/torsion, nuchal/body cords, short cords (<40 cm), long cords (>70 cm), velamentous, marginal or furcate insertion, funisitis, and Wharton’s jelly abnormalities. The studies in this review included retrospective cohort studies, observational studies, case series, and case reports, as long as sufficient data were present. The PICO framework for this review was performed with the following parameters: Population—Singleton pregnancies with reported umbilical vascular thrombosis; Exposure—Umbilical cord abnormalities; Comparator—When available, pregnancies without reported UCAs or without thrombosis; Outcome—Occurrence and characteristics of umbilical arterial thrombosis, umbilical venous thrombosis, or UVTE and associated fetal or neonatal outcomes.

### 2.2. Information Sources

**Figure 1:**
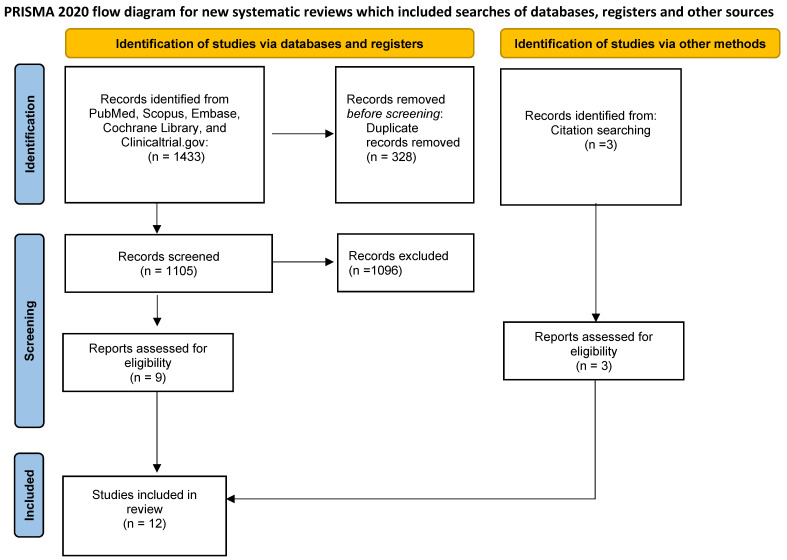
Flowchart of the search strategy for umbilical cord thrombosis.

The Preferred Reporting Items for Systematic Review and Meta-analysis (PRISMA) statement guidelines were followed (Table S1) [17]. The search strategy for umbilical cord thrombosis-related literature is shown in Figure 1. The study protocol was registered on the International Prospective Register of Systematic Reviews (CRD420251087525). The following databases were then surveyed: PubMed, Scopus, Embase, Cochrane Library, and Clinicaltrial.gov.

### 2.3. Search Strategy

On 14 October 2025, five electronic databases (PubMed, Scopus, Embase, Cochrane Library and ClinicalTrial.gov) were searched adopting the following MeSh terms and keywords like “umbilical cord insertion abnormalities” AND “ umbilical cord abnormalities” AND “thrombosis” AND “umbilical artery thrombosis” or “umbilical vascular thromboembolism” (UVTE). No time limit was determined.

### 2.4. Study Selection

The selected studies were analyzed by two reviewers (M.D.S. and E.B.), excluding duplicates and those records that did not consider UCAs associated with thrombosis. The inclusion criteria were: (1) single pregnancy, (2) intrauterine thrombosis in pregnancy, (3) umbilical cord insertion abnormalities or UCAs, (4) umbilical artery thrombosis (UAT) or umbilical vascular thromboembolism (UVTE). Studies that did not meet these criteria were excluded, as well as retrospective case studies and case reports with incomplete clinical data. Disagreements were resolved by a third reviewer (MLV). Additional articles were identified from other sources and were obtained by backward reference screening of eligible articles and relevant reviews.

### 2.5. Data Extraction

We extracted the type of study, first author, year of publication, country, site of UAT or UVTE, total number of patients, and types of UCAs. The consistency of data extraction after completing the entire process was then checked. Disagreements between the authors (M.D.S. and E.B.) were resolved by consulting a third author (M.L.V.).

### 2.6. Risk of Bias Assessment

Two authors (M.D.S. and E.B.) independently assessed the risk of bias and the methodological quality with a modified Newcastle Ottawa Scale [18] (Table S1) and independently rated the study’s quality (Table S1). Any discrepancies between reviewers were resolved by a third reviewer (M.L.V.).

### 2.7. Outcome Measures and Data Synthesis

The primary outcome was the association between UCAs and UVTE. The secondary outcomes included the type of UCA, the site of thrombosis and fetal or neonatal outcome when reported. Findings were synthesized descriptively and organized by type of cord abnormality and thrombosis site. Meta-analysis was not feasible because of heterogeneity in study design, population, diagnostic criteria, outcome definitions, and reporting formats, as well as limited denominator and effect-estimate data.

## 3. Results

### 3.1. Study Selection

The search identified 1433 records. After removing duplicates, 1105 records were screened, and 12 articles were included in the systematic review [10,19,20,21,22,23,24,25,26,27,28,29]. Disagreements about eligibility (M.D.S. and E.B.) were resolved by a third author (M.L.V.).

### 3.2. Study Characteristics and Synthesis of the Results

The 12 articles comprised 3 retrospective cohort studies, 3 case series [22,23,25] and 6 case reports [10,19,20,21,24,26,27,28,29]. The publication years ranged from 1983 to 2025. A total of 126 cases of UVTE were identified, among which 84 cases were of UCAs. To reduce overlap and ambiguity, abnormalities were grouped by abnormal cord length (long or short cord), abnormal cord coiling or torsion (hypercoiling, twisting, and/or stricture), abnormal insertion (velamentous, marginal, or furcate), mechanical compression events (true knots, nuchal/body cord, and/or prolapse), inflammatory lesions (funisitis), and abnormal Wharton’s jelly. Table 1 summarizes the publication year, country of origin, study design/type (case series, case report, or observational study), number of victims enrolled, and gross UCA assessment type.

Most UVTE cases were discovered postpartum upon macroscopic and histological examination. Devlieger et al. (1983) identified one case of UAT associated with a short umbilical cord (<40 cm) [27]. Sato et al. (2006) report 11 cases of UAT had occlusive thrombi of one umbilical artery, 9 cases had UCAs, including long cords, peripheral cord insertions, short cords with twists, and funisitis [19]. Heifetz et al. (1988) found that the thrombosis of the umbilical vein occurs more frequently than thrombosis of one or both umbilical arteries, since umbilical vein thrombosis was present in 90% of their case series of 52 victims of UVTE [10]. UVTE occurred alone in 62% of cases, while UTA occurred in one or both umbilical arteries in 23% of cases [10]. Only 15% of the victims had arterial thrombosis without venous thrombosis. Among 52 cases of UVTE, 39 had UCAs [10], including 10 marginal insertions of the umbilical cord, 8 excessively short cords, 2 excessively long cords, 3 umbilical cord true knots, 8 funisitis and 14 strictures [10]. Avagliano et al. (2010) found 32 umbilical vessel thromboses from a total of 317 consecutive autopsies [20]; 13 out of the 32 total cases showed UCAs, among which were 2 nuchal cords, 5 hypercoiling, 1 marginal cord insertion, 4 true knots, 1 prolapse, 1 funisitis, 1 case of strictures, and 3 Wharton’s jelly abnormalities [20]. Klaritsch et al. (2008) report a case of spontaneous intrauterine UAT in a 28-year-old nulliparous third gravida with intrauterine fetal growth restriction at 32 weeks of gestation, whose umbilical cord was hypercoiled and excessive in length [21]. Shilling et al. (2014) reported a case series including seven fetuses with UAT and six cases with UCAs. The identified abnormalities were four short cords (<40 cm) and two long cords (>70 cm) [25]. Lutfallah et al. (2018) described a case of an elongated, hypercoiled cord with thrombosis of one artery [28]. Wei et al. (2021) conducted a retrospective study with eight cases of UAT, among which three were umbilical cord hypercoiling, one was a case of strictures, one was velamentous insertion, and one was a true knot [22]. A series of 10 UAT cases in the third trimester pregnancy was reported by Zhu et al. (2021), among which intrauterine fetal growth restriction was found in 5 cases [23]. UCAs were detected in six cases and represented by two instances of hypercoiling, one excessively short cord, one excessively long cord and two instances of funisitis [23]. Li et al. (2024) reported one case of UAT [29].

Devlieger et al. (1983) identified one UAT case associated with a short umbilical cord (in association with a hypercoiled and marginal insertion [27]). Ferretti et al. (2025) reported a case of umbilical furcate insertion into the chorionic plate associated with two tight knots and a partial umbilical vein thrombosis [24]. Ding et al. (2025) described, in a single-case report, an umbilical cord that demonstrated hypercoiling and excessive length (>70 cm), leading to arterial thrombosis [26]. The aforementioned data and all other umbilical cord conditions are summarized in Table 2 and Table 3.

### 3.3. Quality Assessment

Seven studies showed a low risk of bias [10,19,20,22,23,25], and six case reports showed a low risk of bias in three domains [21,24,26,27,28,29]. The risk of bias is reported in Table 4. Given that case reports demonstrate limited generalizability and the absence of comparison sets, and cannot estimate incidence or relative risk, the overall level of certainty of the evidence remains low to moderate, so the conclusions should be interpreted as hypothesis-generating rather than causal.

## 4. Discussion

This systematic review focusing on the relationship between the UCAs and the UVTE included 12 articles reporting 126 UVTE and 84 UCAs. The UCAs mostly found in association with UVTE were as follows: 16 cases of stricture, 14 cases of hypercoiling, 16 too-short cords (<40 cm), 11 too-long cords (>70 cm), 5 velamentous or furcate cord insertions, 12 nuchal cord insertions, 13 funisitis, 11 true knots, and 3 Wharton’s jelly abnormalities.

These results highlight the need for prenatal monitoring and pathological evaluation of the umbilical cords. UCAs may contribute to unexpected stillbirth, especially in the absence of other maternal or fetal complications. Although umbilical cord diameter and placental alterations, such as furcate insertion, have received less attention, in the absence of congenital malformation or maternal infection, umbilical hypercoiling may compromise blood supply [30] and UCAs may contribute to unexplained stillbirths [31].

Common diagnostic criteria or prenatal assessments are often inadequate, even in high-income countries [8]. These conditions may be underdiagnosed and identified only after delivery. Vascular occlusion due to venous or arterial thrombosis is mostly associated with UCAs, such as true knots and nuchal cord [3]. UVTE, particularly UAT, is rare, compared to instances with knots and a nuchal cord [3].

According to Sato et al., UAT can occur in 0.025% of pregnancies [19]. Heifetz et al. identified 5 cases out of 11,238 deliveries (0.045%) and 3 cases out of 30,000 autopsies (0.001%) [10]. Wei et al. report eight cases of UAT corresponding to a rate of 0.8% [22]. Avagliano et al. detected 32 cases (10.1%) of umbilical vessel thrombosis in 317 fetal autopsies [20]. Approximately 70% of cases involved isolated thrombosis, 20% involved combined venous and arterial thrombosis, and 10% involved isolated arterial thrombosis (Table 2).

Although umbilical cord thrombosis is associated with anatomic, fetal or maternal conditions, its cause remains unclear. According to Virchow’s hypothesis, Avagliano et al. proposed a relevant role of endothelial damage caused by intrauterine infection [20]. This process may begin with fetal inflammation, diapedesis and neutrophil transmigration, followed by Wharton jelly infiltration and endothelial activation. Subsequent vascular smooth muscle lysis may ultimately lead to thrombosis.

However, most cases of umbilical cord thrombosis are associated with UCAs. Hypercoiling, strictures, too-long or too-short cords, knots, deficiency of Wharton’s jelly and abnormal cord insertion may reduce the blood flow and promote stasis and thrombosis. According to this systematic review, pregnancy complications are pathogenically linked to UCAs and may contribute to umbilical cord thrombosis. The coiling of the umbilical cord makes it flexible and sturdy, which makes it resistant to external factors, but it can also impair blood flow. Abnormal umbilical cord coiling was linked to negative perinatal outcomes [32].

Among the other UCAs analyzed in the present systematic review, there were 16 cases with too-short cords (<40 cm) and 11 cases of too-long cords (>70 cm). Excessively long or short umbilical cords may be predisposed to vascular stasis, as well as velamentous insertion, marginal insertion, umbilical cord entanglement, and excessively twisted cords [33]. Nevertheless, according to Krakowiak et al. (2004), short cords were associated with SGA births, hypoxic–ischemic encephalopathy, and infant death [34]. Abnormal abdominal cord insertion, like true knots and hypercoiling, had an increased risk of intermittent or chronic hypoxia secondary to mechanical obstruction of blood flow [30].

The umbilical cord itself may also become compromised with a predisposition to partial or total thrombosis of the umbilical vein. Velamentous insertion increases the susceptibility of the umbilical vessels to rupture or compression [35]. However, this condition can be detected using colour Doppler imaging, especially during the first trimester of pregnancy [35]. Of course, the diagnosis and management of UCAs are better afforded in high-resource settings, where increased levels of ultrasonography and perinatal pathology are found.

Advanced imaging methods, including 3D ultrasound or 3D Doppler, warrant further refinement for detecting umbilical abnormalities. The site of placental attachment is as important as the degree of cord coiling. 3D Doppler may identify high-risk fetuses, but additional studies are needed. The limitations of this study are mainly represented by the small sample size. Non-uniform antenatal protocol and different pathological evaluations are present. Variability in study design, outcomes, and diagnostic methods excluded a quantitative analysis. Despite these limitations, our review’s findings evidenced the necessity to pay more attention during prenatal ultrasound examination for cord insertion, coiling index, and vascular Doppler, particularly in high-risk pregnancies (gestational diabetes or polyhydramnios of unknown origin), due to the higher risk factors for hypercoiling conditions and umbilical thrombosis. Macroscopic and microscopic examinations of the umbilical cords and placentas in cases of stillbirth are advisable. Standard criteria for the diagnosis and reporting of UCAs need to be established. Furthermore, the maternal mental health status of these patients should be explored [36,37]. Counselling of parents regarding the implications and possible pathways of UCA management represents an additional challenge [38]. A subsequent pregnancy could present an additional risk for the onset of perinatal and postpartum depression [39,40,41]. Large multicenter prospective studies are needed to evaluate the routine assessment of the umbilical cord and its possible impact on perinatal morbidity and mortality.

## 5. Conclusions

This systematic review shows an association between UCAs and umbilical cord thrombosis, particularly in cases involving abnormal coiling, cord strictures, abnormal cord length, true knots, and abnormal placental insertion. UCAs and umbilical cord thrombosis may contribute to unexpected stillbirths in the absence of congenital malformation or maternal infection. These abnormalities are commonly identified in the post-partum period, after a macroscopical and microscopical examination of the fetus and placenta. This highlights the importance of the prenatal umbilical cord evaluation to support a prompt diagnosis and management of high-risk pregnancies and to support families who have experienced stillbirths.

## Figures and Tables

**Table 1:** Characteristics of the included studies.

First Author Publication Year	Country	Study Type	Period of Enrolment	No. of Victims	Umbilical Cord, Gross Abnormalities Assessed
Devlieger H et al. [27]	Belgium	Case report	1983	1	Short cord <40 cm
Sato Y. et al. 2006 [19]	USA	Retrospective Cohort study	1976–2005	11	Abnormal insertion, long cord >70 cm, short cord <35 cm, cord twist
Heifetz SA et al. 1988 [10]	USA	Retrospective Observational study	1945–1987	52	Nuchal coils, stricture/torsion, hematoma, true knot, peripheral insertion, long cord >70 cm, short cord <40 cm, abnormality of Wharton’s jelly
Avagliano L. et al. 2010 [20]	Italy	Retrospective observational study	1997–2007	32	Site of TUV, true knot, funisitis, hypercoiled cords, marginal insertion, nuchal/body cord, Wharton jelly hemorrhage
Klaritsch P. et al. 2008 [21]	Austria	Case report	2007	1	Long cord >70 cm, cord twist, placenta’s weight, number of vessels
Shilling C et al. 2014 [25]	Ireland	Case series	1999–2012	7	Long cord >70 cm, short cord <40 cm
Lutfallah F. et al. 2018 [28]	France	Case report	2018	1	Elongated cord, hypercoiling
Wei J. et al. 2021 [22]	China	Case series	2018–2020	8	Hypercoiling, true knot, velamentous cord insertion.
Zhu Y. et al. 2021 [23]	China	Case series	2015–2019	10	Irregular length of the umbilical cord, narrowed cord with hypercoiling, swollen cord with Wharton’s jelly deficiency, placenta velamentous and umbilical infarction, funisitis
Li J et al. 2024 [29]	China	Case Report	2024	1	Hypercoiling, nuchal cord
Ferretti et al. 2025 [24]	Italy	Case report	2025	1	Furcate insertion into the chorionic plate associated with the two tight knots
Ding W et al. 2025 [26]	China	Case Report	2025	1	Long cord >70 cm, hypercoiling

**Table 2:** Sites and frequency of umbilical cord gross abnormalities in UVTE. UVTE: Umbilical vascular thromboembolism, UA: umbilical artery, UV: umbilical vein.

First Author Publication Year	Country	No. of Patients	Site of UVTE	No. of Umbilical Cord Gross Abnormalities Assessed/Total of Cases with UVTE (%)
Devlieger H. et al., 1983 [27]	Belgium	1	UA	1/1 (100%)
Sato Y. et al., 2006 [19]	USA	11	UA	9/11 (81.8%)
Heifetz S.A. et al., 1988 [10]	USA	52	UA and UV	47/52 (91%)
Avagliano L. et al., 2010 [20]	Italy	32	UA and UV	13/32 (40.6%)
Klaritsch P. et al., 2008 [21]	Austria	1	UA	1/1 (100%)
Shilling C. et al., 2014 [25]	Ireland	7	UA	6/7 (85%)
Lutfallah F. et al., 2018 [28]	France	1	UA	1/1 (100%)
Wei J. et al., 2021 [22]	China	8	UA	4/8 (50%)
Zhu Y. et al., 2021 [23]	China	10	UA and UV	7/10 (70%)
Li J. et al., 2024 [29]	China	1	UA	1/1 (100%)
Ferretti et al., 2025 [24]	Italy	1	UV	1/1 (100%)
Ding W et al., 2025 [25]	China	1	UA	1/1 (100%)

**Table 3:** Umbilical cord gross abnormalities outcomes.

First Author Publication Year	No. of Umbilical Cord Gross Abnormalities Assessed/Total of Cases with UVTE	Nuchal Cord	Umbilical Cord Hypercoiling	Velamentous or Furcate Cord Insertion	Excessively Short Cord <40 cm	Excessively Long Cord >70 cm	True Knot	Prolapse	Funisitis	Strictures	Wharton’s Jelly Hemorrhage/Abnormality
Devlieger H et al. 1983 [27]	1/1	N/A	N/A	N/A	1 (100%)	N/A	N/A	N/A	N/A	N/A	N/A
Sato Y. et al. 2006 [19]	9/11	N/A	N/A	2 (18%)	2 (18%)	3 (27%)	N/A	N/A	2 (18%)	N/A	N/A
Heifetz SA et al. 1988 [10]	47/52	9 (17%)	N/A	N/A	8 (15.3%)	2 (3.8%)	3 (5.7%)	0	8 (15.3%)	14 (26.9%)	0
Avagliano L. et al. 2010 [20]	13/32	2 (6.2%)	5 (18.7%)	1 (3.1%)	N/A	N/A	4 (12.5%)	1 (3.1%)	1 (3.1%)	1 (3.1%)	3/15 (9.3%)
Klaritsch P. et al. 2008 [21]	1/1	N/A	1 (100%)	N/A	N/A	1 (100%)	N/A	N/A	N/A	N/A	N/A
Shilling C et al. 2014 [25]	6/7	N/A	N/A	N/A	4 (57%)	2 (33%)	N/A	N/A	N/A	N/A	N/A
Lutfallah F. et al. 2018 [28]	1/1	N/A	1 (100%)	N/A	N/A	1 (100%)	N/A	N/A	N/A	N/A	N/A
Wei J. et al. 2021 [22]	4/8	N/A	3 (37.5%)	1 (12.5%)	N/A	N/A	1 (12.5%)	N/A	N/A	1 (12.5%)	N/A
Zhu Y. et al. 2021 [23]	7/10	N/A	2 (20%)	1 (10%)	1 (10%)	1 (10%)	N/A	N/A	2 (20%)	N/A	N/A
Li J et al. 2024 [29]	1/1	1 (100%)	1 (100%)	N/A	N/A	N/A	N/A	N/A	N/A	N/A	N/A
Ferretti et al. 2025 [24]	1/1	N/A	NA	1 (100%)	N/A	N/A	1 (100%)	N/A	N/A	N/A	N/A
Ding W et al. 2025 [26]	1/1	N/A	1 (100%)	N/A	N/A	1 (100%)	N/A	N/A	N/A	N/A	N/A

N/A: not available.

**Table 4:** Risk of bias assessment.

First Author Publication Year	Study Design and Sample Representativeness	Sampling Technique	Description of the Umbilical Cord Abnormalities	Quality of Population Description	Incomplete Outcome Data	Total Score
Devlieger H et al. 1983 [27]	-	-	★	★	★	★★★
Sato et al. 2006 [19]	★	★	★	★	★	★★★★★
Heifetz SA et al. 1988 [10]	★	★	★	★	★	★★★★★
Avagliano L. et al. 2010 [20]	★	★	★	★	★	★★★★★
P. Klaritsch et al. 2008 [21]	-	-	★	★	★	★★★
Shilling C et al. 2014 [25]	★	★	★	★	★	★★★★★
Lutfallah F. et al. 2018 [28]	-	-	★	★	★	★★★
Wei et al. 2021 [22]	★	★	★	★	★	★★★★★
Zhu et al. 2021 [23]	★	★	★	★	★	★★★★★
Li J et al. 2024 [29]	-	-	★	★	★	★★★
Ferretti et al. 2025 [24]	-	-	★	★	★	★★★
Ding W et al. 2025 [26]	-	-	★	★	★	★★★

Abbreviation: ★, criterion met.

## Data Availability

No new data were created or analyzed in this study.

## Supplementary Materials

The following supporting information can be downloaded at: https://www.mdpi.com/article/10.3390/clinpract16050092/s1, Table S1: PRISMA 2020 checklist [42].

## Abbreviations

The following abbreviations are used in this manuscript:

UCAs	Umbilical cord abnormalities
UVTE	Umbilical vascular thromboembolism
UAT	Umbilical artery thrombosis
PRISMA	Preferred Reporting Items for Systematic Review and Meta-analysis
